# Artificial intelligence-based analysis of lower limb muscle mass and fatty degeneration in patients with knee osteoarthritis and its correlation with Knee Society Score

**DOI:** 10.1007/s11548-024-03284-y

**Published:** 2024-11-03

**Authors:** Kohei Kono, Tomofumi Kinoshita, Mazen Soufi, Yoshito Otake, Yuto Masaki, Keisuke Uemura, Tatsuhiko Kutsuna, Kazunori Hino, Takuma Miyamoto, Yasuhito Tanaka, Yoshinobu Sato, Masaki Takao

**Affiliations:** 1 Department of Orthopaedic Surgery, Ehime University Graduate School of Medicine, 454 Shitsukawa, Toon, Ehime 790-0295 Japan; 2 Division of Information Science, Graduate School of Science and Technology, Nara Institute of Science and Technology, Ikoma, Japan; 3 Department of Orthopaedic Medical Engineering, Graduate School of Medicine, Osaka University, Suita, Japan; 4 Department of Orthopedic Surgery, Nara Medical University, Kashihara, Japan

**Keywords:** Artificial Intelligence, Muscle mass, Muscle fatty degeneration, Knee osteoarthritis, Patient-reported outcome measure

## Abstract

**Purpose:**

Lower-limb muscle mass reduction and fatty degeneration develop in patients with knee osteoarthritis (KOA) and could affect their symptoms, satisfaction, expectation and functional activities. The Knee Society Scoring System (KSS) includes patient reported outcome measures, which is widely used to evaluate the status of knee function of KOA. This study aimed to clarify how muscle mass and fatty degeneration of the lower limb correlate with the KSS in patients with KOA.

**Methods:**

This study included 43 patients with end-stage KOA, including nine males and 34 females. Computed tomography (CT) images of the lower limb obtained for the planning of total knee arthroplasty were utilized. Ten muscle groups were segmented using our artificial-intelligence-based methods. Muscle volume was standardized by dividing by their height squared. The mean CT value for each muscle group was calculated as an index of fatty degeneration. Bivariate analysis between muscle volume or CT values and KSS was performed using Spearman’s rank correlation test. Multiple regression analysis was performed, and statistical significance was set at *p*  < 0.05.

**Results:**

Bivariate analysis showed that the functional activity score was significantly correlated with the mean CT value of all muscle groups except the adductors and iliopsoas. Multiple regression analysis revealed that the functional activities score was significantly associated with the mean CT values of the gluteus medius and minimus muscles and the anterior and lateral compartments of the lower leg (β = 0.42, *p* = 0.01; β = 0.33, *p* = 0.038; and β = 0.37, *p* = 0.014, respectively).

**Conclusion:**

Fatty degeneration, rather than muscle mass, in the lower-limb muscles was significantly associated with functional activities score of the KSS in patients with end-stage KOA. Notably, the gluteus medius and minimus and the anterior and lateral compartments of the lower leg are important muscles associated with functional activities.

**Supplementary Information:**

The online version contains supplementary material available at 10.1007/s11548-024-03284-y.

## Introduction

Knee osteoarthritis (KOA) is a global leading cause of functional disability. Lower-limb muscle mass reduction and fatty degeneration develop in patients with KOA and could affect their symptom, satisfaction, expectation and functional activities. Muscle dysfunction associated with KOA may be the primary underlying cause of functional disability [1]. Moreover, muscle dysfunction could precede the progression of KOA, leading to severe pain and difficulty in walking [2, 3]. To evaluate the clinical status in KOA, the Knee Society Scoring system (KSS) has been widely used as a patient-reported outcome measure (PROM) for KOA, focusing on individual knee conditions [4]. This PROM allows for easy scoring and assessment of pain, satisfaction, expectations, and functional activity in KOA. Despite the widespread use of this valuable questionnaire, its correlation with muscle dysfunction remains unclear. The quadriceps muscles, which function during the stance and swing phases of walking, play an important role in patients with KOA [5]. Decline in quadriceps muscle strength is associated with walking difficulties and a decline in QOL in patients with KOA, and quadriceps training is an important conservative treatment. The hamstrings, iliopsoas, gluteus maximus and medius, tibialis anterior, and triceps surae are also important for walking [6–11]. However, the extent of lower-limb muscle mass and fatty degeneration and their correlation with PROMs in patients with KOA are unknown.

Computed tomography (CT) or magnetic resonance imaging (MRI) cross-sectional area assessment of muscle mass and fatty degeneration is the gold standard for the image assessment of muscle quality [12–15]. Fatty degeneration of the muscles varies at different cross-sectional levels, even in the same muscle; therefore, its evaluation at a single cross-sectional level might not be accurate [16, 17]. The three-dimensional (3D) structure of the muscles varies for each muscle, and the conventional method of evaluating the single-plane cross-sectional area cannot accurately reflect the volume and shape of each muscle [18]. The muscle mass and fatty degeneration are preferably evaluated three dimensionally; however, the segmentation of multiple cross-sections is labor-intensive and limits the number of participants and muscles. To solve this problem, artificial intelligence (AI)-based muscle segmentation on CT or MRI has recently been applied for the 3D evaluation of multiple muscles [19, 20].

We developed an AI-based muscle segmentation method for CT-based muscle assessment to investigate the relationship between lower-limb muscle conditions and PROMs in various diseases [20]. Patients with KOA exhibit significant muscle impairments in the quadriceps, hamstrings, and hip muscles compared to age-matched controls [21, 22]. Furthermore, biomechanical analysis of patients with KOA revealed altered movement patterns and muscle activation in the lower limbs, including the hip and lower legs, during functional tasks [23–25]. Based on these findings, we hypothesized that muscle degeneration occurs not only in the thigh muscles, but also in the hips and lower legs of patients with KOA and affects the KSS. This study aimed to examine muscle mass and fatty degeneration throughout the lower limbs of patients with KOA using an AI-based method, clarify their relationship with the KSS, and identify the muscles that need strengthening.

## Methods

Out of 111 patients with KOA who underwent total knee arthroplasty (TKA) at our institution between April 2021 and May 2022, 56 patients agreed to participate and completed a questionnaire. After applying the exclusion criteria described below, 43 patients were included in this cross-sectional study. This study was approved by the Institutional Review Board of our institution (IRB number 2103009). The exclusion criteria were (1) a lack of preoperative CT images; (2) a treatment history of trauma, infection, tumor, or osteoarthritis other than the knee in the affected limb; and (3) a lack of PROMs. Table 1 shows the patient characteristics. All knees were classified as Kellgren–Lawrence (KL) grade 4 [26]. The clinical status of the knee was evaluated using the KSS revised in 2011 [4], a commonly used PROM for patients with KOA. The subjective component of the KSS is a self-administered questionnaire comprising four subscales: symptoms, patient satisfaction, patient expectations, and functional activities.

**Table 1:** Patient characteristics

		Mean ± SD	Range
Sex		34 Females, 9 males	
Age (year)		77.4 ± 6.8	57–89
Body Mass Index (kg/m^2^)		25.6 ± 3.8	18.7–36.9
HKA (°)		10.0 ± 7.1	−8–30
KSS	Symptoms	8.4 ± 3.8	2–17
	Patient satisfaction	13.4 ± 4.3	2–22
	Patient expectation	13.9 ± 1.5	10–15
	Functional activities	37.7 ± 16.3	2–65

*SD* standard deviation, *HKA* hip-knee-ankle angle, *KSS* 2011 Knee Society score, symptoms symptom score of KSS, Patient satisfaction patient satisfaction of KSS, Patient expectation patient expectation of KSS, Functional activities functional activities of KSS

CT images were obtained for preoperative planning using a 320-row multidetector CT system (Aquilion ONE GENESIS Edition, Canon Medical Systems Inc., Otawara, Japan) based on the following protocol: tube voltage, 120 kV; tube current, 50–520 mA; helical pitch, 65.0; slice thickness, 1.00 mm; and X-ray tube rotation speed, 0.5 s. The area from the 10th thoracic vertebra to the toes was included in the imaging range.

**Fig. 1:**
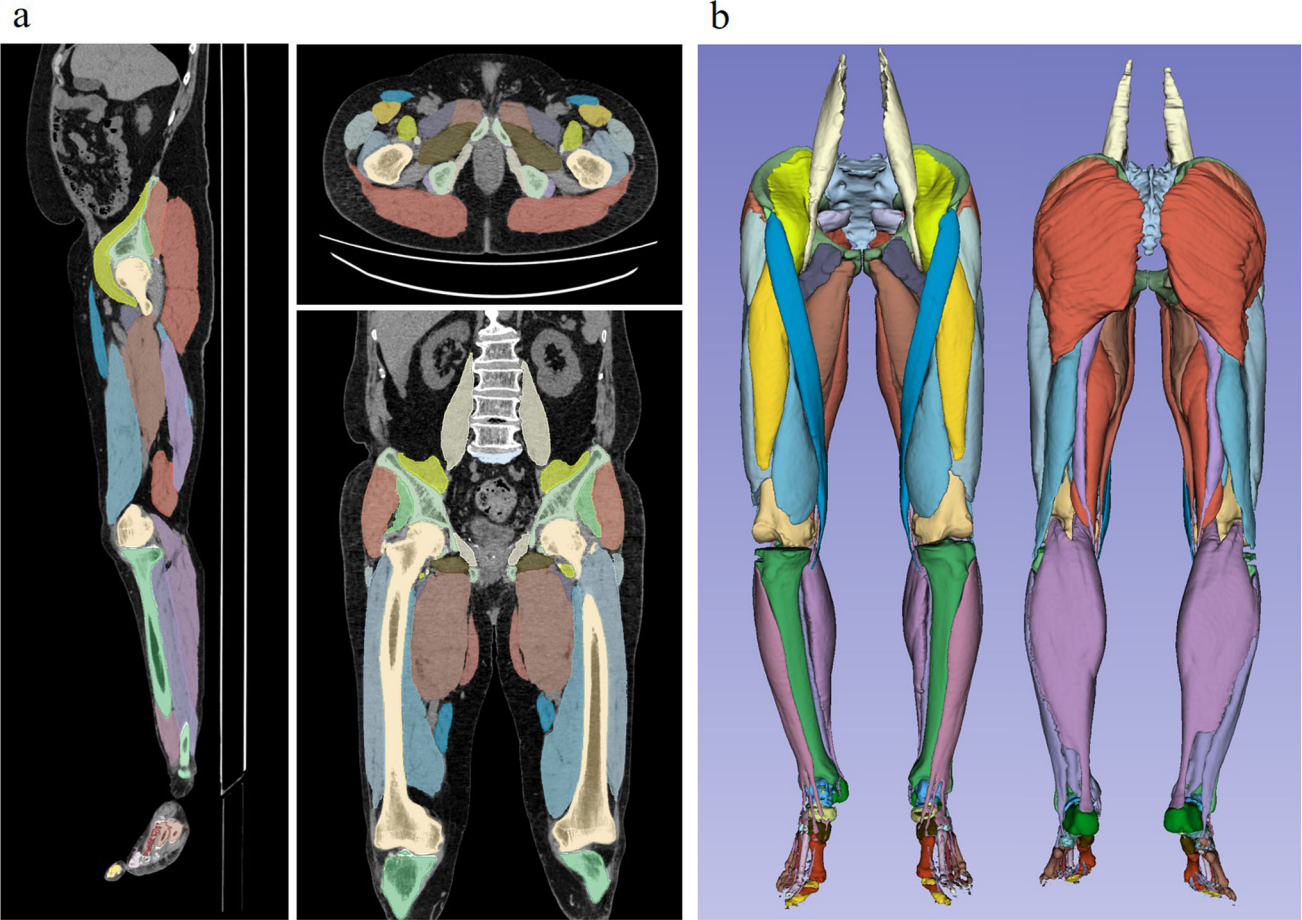
Artificial intelligence-based segmentation model of a patient with knee osteoarthritis. **a** Precise segmentation images of each muscle group. **b** Three-dimensional skeletal muscle model reconstructed from segmentation images

A deep learning-based AI model built from a fully convolutional neural network with a U-Net architecture (Bayesian U-Net), which has a high reliability in bone and muscle assessment in hip-to-knee CTs [19], was used in this study. The muscles were divided into 10 groups according to their function: gluteus maximus, gluteus medius and minimus, iliopsoas, adductors, quadriceps, hamstrings, anterior compartment, lateral compartment, deep posterior compartment, and superficial posterior compartment muscles of the lower leg. AI segmentation models were trained and validated on the CT images used in this study. Two researchers created ground-truth labels of the target muscles on a subset of 34 cases based on an active learning approach [19]. A cross-validation experiment was conducted to verify the accuracy of the AI models. To assess the muscle mass, each muscle volume was divided by the height square (cm^3^/m^2^) and defined as the standardized muscle volume. The average CT value (Hounsfield Unit; HU) for each segmented muscle group was calculated as an index of fatty muscle degeneration (Fig. 1). Attenuation of CT values is reportedly associated with skeletal muscle lipid content [27].

### Statistical analysis

Normality was assessed using the Shapiro–Wilk test. The CT values for each muscle and KSS for each subscale were not normally distributed; however, the muscle volumes showed a normal distribution. Bivariate analysis between the KSS subscales and standardized muscle volume or mean CT values was performed using Spearman’s rank correlation coefficient tests. Multiple linear regression analysis was performed using the muscle groups that showed statistically significant correlations with the KSS subscales as independent variables. Confounding factors such as age, sex, and body mass index were included as adjustment variables. Statistical significance was set at *p* < 0.05. All statistical analyses were performed using SPSS software version 28.0 (IBM Japan, Tokyo, Japan).

## Results

### AI model validation

The segmentation accuracy (Dice coefficient) of the muscles in the cross-validation experiment (34 cases) using the AI model and the average surface error were 0.958 ± 0.020 and 0.560 ± 0.770 mm, respectively. Accuracy of the muscle volume and mean HU measurements were 2.690 ± 2.870% and 0.647 ± 0.707 HU, respectively.

### Muscle measurements

Table 2 presents the standardized muscle volume and mean CT value of each muscle group on the affected side of patients with KOA. The no-muscle group showed a significant correlation between standardized muscle volume and each subscale score of the KSS (Table 3). In contrast, the mean CT values of several muscle groups significantly correlated with the symptom and functional activity subscale scores. The symptom subscale score significantly correlated with the mean CT value of the lower-leg anterior compartment. The functional activity subscale score was significantly correlated with the mean CT values of all muscle groups except the adductors and iliopsoas (Table 4). In the multiple linear regression analysis, the functional activity subscale score was significantly associated with the mean CT values of the gluteus medius and minimus and the anterior and lateral compartments of the lower leg (Table 5).

**Table 2:** Muscle mass and the degree of fatty degeneration on the affected side assessed using standardized muscle volume and CT values

	Standardized muscle	CT values
	Volume (cm^3^/m^2^)	HU
	Mean ± SD	Mean ± SD
Gluteus maximus	272.3 ± 52.0	16.9 ± 12.1
Gluteus medius and minimus	123.8 ± 17.0	25.6 ± 10.4
Iliopsoas	82.2 ± 15.9	41.0 ± 5.0
Adductor muscles	277.3 ± 48.4	35.5 ± 6.2
Quadriceps	340.3 ± 64.8	40.5 ± 6.9
Hamstrings	197.5 ± 33.0	29.9 ± 10.4
Anterior compartment	75.5 ± 10.7	41.0 ± 7.0
Lateral compartment	38.7 ± 8.2	43.4 ± 7.1
Deep posterior compartment	89.1 ± 14.7	36.9 ± 8.1
Superficial posterior compartment	208.8 ± 46.0	34.6 ± 10.3

*SD* standard deviation, *CT* computed tomography, *HU* Hounsfield Unit

**Table 3:** Correlation between KSS and standardized muscle volume (cm^3^/m^2^)

	Symptoms		Patient satisfaction		Patient expectation		Functional activities	
	ρ	*p-*value	ρ	*p-*value	ρ	*p-*value	ρ	*p-*value
Gluteus maximus	−0.124	0.429	−0.194	0.212	0.231	0.137	−0.064	0.683
Gluteus medius and minimus	0.096	0.541	−0.29	0.059	0.13	0.406	−0.153	0.326
Iliopsoas	0.241	0.119	−0.158	0.311	−0.044	0.779	0.139	0.375
Adductor muscles	0.017	0.914	−0.204	0.189	0.257	0.097	0.11	0.484
Quadriceps	0.142	0.365	0.003	0.987	0.175	0.262	0.001	0.997
Hamstrings	−0.101	0.521	−0.261	0.091	0.174	0.263	0.036	0.818
Anterior compartment	−0.03	0.848	−0.275	0.075	0.162	0.301	−0.233	0.133
Lateral compartment	−0.201	0.196	−0.121	0.439	0.121	0.438	−0.248	0.109
Deep posterior compartment	0.07	0.656	−0.066	0.672	0.162	0.301	−0.031	0.845
Superficial posterior compartment	−0.92	0.558	−0.145	0.352	−0.004	0.981	−0.047	0.763

2011 Knee Society score, KSS; Symptoms, symptom score of KSS; Patient satisfaction, patient satisfaction of KSS; Patient expectation, patient expectation of KSS; Functional activities, functional activities of KSS

**Table 4:** Correlation between KSS and fatty muscle degeneration (HU)

	Symptoms		Patient satisfaction		Patient expectation		Functional activities	
	ρ	*p-*value	ρ	*p-*value	ρ	*p-*value	ρ	*p-*value
Gluteus maximus	0.13	0.406	−0.065	0.68	−0.135	0.389	0.448	0.003**
Gluteus medius and minimus	0.04	0.799	−0.112	0.474	−0.009	0.952	0.53	< 0.001**
Iliopsoas	0.174	0.265	−0.131	0.404	−0.115	0.464	0.113	0.47
Adductor muscles	0.185	0.236	−0.266	0.085	0.007	0.965	0.262	0.09
Quadriceps	0.28	0.069	−0.089	0.57	−0.144	0.356	0.328	0.032*
Hamstrings	0.08	0.611	−0.127 ara>	0.418	−0.23	0.885	0.433	0.004**
Anterior compartment	0.32	0.037*	−0.005	0.977	−0.168	0.282	0.464	0.002**
Lateral compartment	0.279	0.07	0.023	0.885	−0.142	0.362	0.511	< 0.001**
Deep posterior compartment	0.239	0.123	−0.113	0.469	−0.108	0.49	0.459	0.002**
Superficial posterior compartment	0.247	0.11	−0.087	0.577	−0.131	0.401	0.395	0.009**

2011 Knee Society score, KSS; Symptoms, symptom score of KSS; Patient satisfaction, patient satisfaction of KSS; Patient expectation, patient expectation of KSS; Functional activities, functional activities of KSS; HU, Hounsfield Unit

^*^, *p* < 0.05; **, *p* < 0.01

**Table 5:** Multiple linear regression analysis to evaluate the correlation between muscle fatty degeneration and the functional activity score of the KSS

	Muscle segmentation	β	95%CI	*p-*value
Functional activities	Gluteus medius and minimus	0.42	0.17–1.171	0.01*
	Anterior compartment of the lower leg	0.33	0.04–1.48	0.038*
	Lateral compartment of the lower leg	0.37	0.18–1.51	0.014*

KSS, the 2011 Knee Society score; Functional activities, functional activities of KSS; *β* standard regression coefficient; *CI* confidence interval

*p < 0.05; **p < 0.01

## Discussion

In this study, a 3D analysis of muscle mass and fatty degeneration of the entire lower limb in patients with end-stage KOA was performed using an AI-based muscle segmentation method to clarify its correlation with the KSS. This study had three critical findings. First, fatty degeneration, rather than muscle mass, of the lower-limb muscles, except for the adductors and iliopsoas muscles, was significantly associated with functional activity in bivariate analysis. Second, fatty degeneration of the gluteus medius and minimus and the anterior and lateral compartments of the lower leg significantly correlated with decreased functional activity in multiple regression analyses. Third, the symptom score was correlated with the degree of fatty degeneration of the anterior compartment of the lower leg in bivariate analysis. Fatty degeneration of muscles means loss of contractile elements, greatly impacting the symptoms and physical activities rather than muscle mass. The quantitative evaluation of muscle mass and fatty muscle degeneration in a previous study utilized CT or MRI to evaluate the cross-sectional area [12–15]. To the best of our knowledge, this is the first study to perform a 3D assessment of muscle mass and fatty degeneration throughout the lower limb and report its correlation with PROMs in patients with KOA.

The activation of the hip abductor muscles, comprising the gluteus medius and minimus, is crucial for maintaining posture during standing and transferring. Therefore, it is natural that the degree of fatty muscle degeneration of the gluteus medius and minimus was correlated with the functional activity subscale score. A meta-analysis reported that strengthening the hip abductor muscle positively impacts knee pain and functional outcomes in patients with KOA [9]. This suggests that fatty degeneration of the gluteus medius and minimus in patients with KOA significantly impacts their physical function. This might be because the progression of knee varus deformity leads to standing and walking postures with hip abduction, which might cause hip abductor dysfunction. In this study, the mean age of the patients was 77.4 ± 6.8 years, which is similar to the mean age (75 years) at TKA reported in joint registry in our country but suggests overlap of sarcopenia exists with muscle deterioration with KOA. Fu et al. reported that patients with sarcopenia had greater gluteus medius and minimus atrophy than those without sarcopenia [28]. The prevalence of sarcopenia has been reported to be approximately 10–30% preoperatively in patients undergoing TKA [29–31]. A longitudinal study reported that a decrease in muscle mass can only explain approximately 6–8% of the decrease in muscle strength because muscle weakness is related to the muscle fatty degeneration due to aging [32].

Fatty degeneration of the lower-leg muscles may also be caused by KOA progression. The peroneus muscle, a component of the lateral compartment of the leg, pronates the ankle joint [33]. Patients with varus knee deformity due to KOA have a pronated ankle position than healthy individuals [34]. Biomechanical studies have shown that patients with KOA with greater foot pronation during gait have reduced loads on the medial knee joint [35]. Therefore, loss of foot pronation due to peroneal muscle dysfunction can exacerbate pain and further movement problems. Further biomechanical research is needed to prove this speculation that the dysfunction of the lateral compartment muscle dysfunction of the lower leg correlated with walking disability in patients with KOA.

The tibialis anterior (TA) muscle, the main muscle of the anterior compartment of the lower leg, is the ankle joint dorsiflexor [33]. Decreased stair-climbing performance in patients with KOA is associated with dorsiflexion torque deficits [10]. In addition, the ankle joint must be dorsiflexed to set the plantar foot on the ground with the ankle joint in a pronation position. As the muscle strength of the TA, which controls such movements, decreases, this may lead to a deterioration in the quality of movement in patients with KOA. Further biomechanical research is needed to prove this speculation that the dysfunction of the anterior compartment muscle dysfunction of the lower leg correlated with decreased stair-climbing performance in patients with KOA.

In previous studies, the central concern was the quadriceps muscle in patients with KOA. The quadriceps muscles in patients with KOA have been reportedly more atrophied and degenerated than those in healthy individuals [12, 13, 15, 36]. Moreover, preoperative quadriceps strength influences postoperative patient satisfaction and physical function after TKA [37, 38]. Notably, several researchers have demonstrated the influence of fatty degeneration of the quadriceps muscle on dysfunction and worsening of symptoms score [13, 36, 39]. Therefore, previous conservative therapies and studies on KOA have focused on the quadriceps muscles. In this study, the degree of fatty muscle degeneration of the quadriceps muscles was correlated with the functional activity score in the bivariate analysis. However, there was no significant correlation between these factors in multiple linear regression analysis. In this study, participants presented with end-stage KOA, and CT evaluation was performed immediately before TKA surgery. Our study may have examined patients who were more symptomatic and had a more advanced decline in activity than those in previous studies. This result also suggests that fatty muscle degeneration throughout the lower limb following fatty quadriceps degeneration reflects decreased physical function in patients with end-stage KOA.

This study has some limitations. First, the number of patients included in this study was small. Only 43 patients were included in this study. Therefore, only a limited number of independent variables were available for multivariate analysis, and the confidence interval was also wider. Sex was not standardized, and the study included nine males; we also performed the same analysis for the female patients only and the results are the same as those of the total cohort (Supplementary Tables 1 to 3). Further study focusing on the sex difference in muscle mass and fatty degeneration in patients with KOA would be necessary. In addition, this was a preliminary study to examine how muscle mass and fatty degeneration, evaluated using a 3D method, correlate with the KSS in patients with KOA. Further studies with larger sample sizes are required. Second, the muscle status of the contralateral limb was not assessed, as the focus was on the impact of the symptomatic lower limb on the KSS. However, most patients exhibit variable levels of osteoarthritic changes on the contralateral side, with or without pain. Therefore, including many patients and evaluating the correlation between bilateral limb muscle conditions and the KSS is necessary. Third, the study did not include a control group. Therefore, it is unclear how KOA is associated with muscle atrophy and fatty degeneration compared with age- and sex-matched healthy individuals. Large-scale studies are required to determine the standard muscle mass and CT values according​​ to age and sex. Finally, participant bias might have occurred in the KSS. The KSS is a questionnaire specific to knee disorders; however, participant bias might have affected the outcomes regardless of the patient intentions. Further studies using objective physical functional indicators such as wearable devices and motion analysis would be necessary.

## Conclusion

Fatty degeneration, rather than muscle mass, in the lower-extremity muscles was significantly associated with the physical functional score of the KSS in patients with end-stage KOA. Notably, the gluteus medius and minimus, and the anterior and lateral compartments of the lower leg, are important muscles associated with decreased physical function. Based on these findings, conventional rehabilitation methods focusing on training the quadriceps muscles in patients with KOA should be reconsidered.

## Supplementary Information

Below is the link to the electronic supplementary material.

Supplementary file1 (DOCX 38 KB)
