# Supplementary material for: Artificial intelligence-based analysis of lower limb muscle mass and fatty degeneration in patients with knee osteoarthritis and its correlation with Knee Society Score
Source: Int J Comput Assist Radiol Surg. 2024 Nov 3;20(4):635–42. doi: 10.1007/s11548-024-03284-y (PMC12034593; doi:10.1007/s11548-024-03284-y)
Supplement: Supplementary file 1 — Supplementary file1 (DOCX 38 KB) [file 11548_2024_3284_MOESM1_ESM.docx]

**Supplemental Table 1.** Correlation between KSS and standardized muscle volume (cm^3^/m^2^) in females

|  | Symptoms | | Patient satisfaction | | Patient expectation | | Functional activities | |
| --- | --- | --- | --- | --- | --- | --- | --- | --- |
|  | ρ | *p-*value | ρ | *p-*value | ρ | *p-*value | ρ | *p-*value |
| gluteus maximus | -0.201 | 0.255 | -0.18 | 0.309 | 0.162 | 0.36 | -0.168 | 0.342 |
| gluteus medius and minimus | 0.04 | 0.824 | -0.152 | 0.359 | 0.068 | 0.702 | -0.334 | 0.053 |
| iliopsoas | 0.108 | 0.543 | -0.265 | 0.311 | -0.116 | 0.512 | -0.104 | 0.559 |
| adductor muscles | -0.047 | 0.792 | -0.196 | 0.267 | 0.087 | 0.623 | -0.077 | 0.663 |
| quadriceps | 0.028 | 0.873 | 0.063 | 0.722 | 0.061 | 0.73 | -0.225 | 0.201 |
| hamstrings | -0.208 | 0.238 | -0.268 | 0.092 | 0.049 | 0.784 | -0.101 | 0.571 |
| anterior compartment | -0.138 | 0.435 | -0.296 | 0.085 | 0.122 | 0.494 | -0.239 | 0.698 |
| lateral compartment | -0.32 | 0.065 | -0.148 | 0.402 | 0.148 | 0.405 | -0.298 | 0.087 |
| deep posterior compartment | 0.008 | 0.963 | -0.137 | 0.439 | 0.116 | 0.513 | -0.157 | 0.375 |
| superficial posterior compartment | -0.169 | 0.338 | -0.127 | 0.474 | -0.064 | 0.721 | -0.097 | 0.586 |

2011 Knee Society score, KSS; Symptoms, symptom score of KSS; Patient satisfaction, patient satisfaction of KSS; Patient expectation, patient expectation of KSS; Functional activities, functional activities of KSS

*, *p* < 0.05; **, *p* < 0.01

**Supplemental Table 2.** Correlation between KSS and fatty muscle degeneration (HU) in females

|  | Symptoms | | Patient satisfaction | | Patient expectation | | Functional activities | |
| --- | --- | --- | --- | --- | --- | --- | --- | --- |
|  | ρ | *p-*value | ρ | *p-*value | ρ | *p-*value | ρ | *p-*value |
| gluteus maximus | 0.085 | 0.633 | -0.181 | 0.307 | -0.256 | 0.08 | 0.34 | 0.049* |
| gluteus medius and minimus | -0.021 | 0.907 | -0.151 | 0.394 | -0.239 | 0.173 | 0.469 | 0.005** |
| iliopsoas | 0.116 | 0.514 | -0.288 | 0.099 | -0.211 | 0.232 | -0.019 | 0.915 |
| adductor muscles | 0.162 | 0.361 | -0.246 | 0.078 | -0.202 | 0.251 | 0.041 | 0.819 |
| quadriceps | 0.328 | 0.058 | -0.131 | 0.462 | -0.264 | 0.267 | 0.165 | 0.351 |
| hamstrings | 0.086 | 0.627 | -0.153 | 0.387 | -0.246 | 0.161 | 0.357 | 0.038* |
| anterior compartment | 0.346 | 0.045* | -0.062 | 0.727 | -0.259 | 0.266 | 0.446 | 0.008** |
| lateral compartment | 0.309 | 0.075 | -0.071 | 0.69 | -0.282 | 0.106 | 0.509 | 0.002** |
| deep posterior compartment | 0.253 | 0.148 | -0.13 | 0.462 | -0.243 | 0.166 | 0.407 | 0.017* |
| superficial posterior compartment | 0.227 | 0.196 | -0.156 | 0.377 | -0.289 | 0.097 | 0.294 | 0.091 |

2011 Knee Society score, KSS; Symptoms, symptom score of KSS; Patient satisfaction, patient satisfaction of KSS; Patient expectation, patient expectation of KSS; Functional activities, functional activities of KSS; HU, Hounsfield Unit

*, *p* < 0.05; **, *p* < 0.01

**Supplemental Table 3.** Multiple linear regression analysis to evaluate the correlation between muscle fatty degeneration and the functional activity score of the KSS in females

|  | Muscle segmentation | β | 95%CI | *p-*value |
| --- | --- | --- | --- | --- |
| Functional activities | gluteus medius and minimus | 0.38 | 0.10-1.23 | 0.002** |
|  | anterior compartment of the lower leg | 0.34 | 0.03-1.66 | 0.043* |
|  | lateral compartment of the lower leg | 0.39 | 0.18-1.66 | 0.017* |

KSS, the 2011 Knee Society score; Functional activities, functional activities of KSS; β, standard regression coefficient; CI, confidence interval

*, *p* < 0.05; **, *p* < 0.01
